# Modifiable psychosocial risk factors for depression among cancer survivors in the United States

**DOI:** 10.1371/journal.pone.0359599

**Published:** 2026-10-21

**Authors:** Philip Baiden, Batholomew Chireh, Judith S. Anagbonu

**Affiliations:** 1 School of Social Work, The University of Texas at Arlington, Arlington, Texas, United States of America; 2 Child Welfare Research Center, The University of Texas at Arlington, Arlington, Texas, United States of America; 3 Department of Health Sciences, College of Health Professions, The University of Texas Rio Grande Valley, Edinburg, Texas, United States of America; Tribhuvan University Institute of Medicine, NEPAL

## Abstract

Depression is highly prevalent among cancer survivors and contributes to poorer quality of life, reduced treatment adherence, and increased morbidity. Functional limitations, loneliness, and inadequate social and emotional support are important psychosocial factors associated with self-reported lifetime diagnosis of major depressive disorder among cancer survivors. Using a cross-sectional study design, this study examined the independent associations of functional limitations, loneliness, and social/emotional support with a self-reported lifetime diagnosis of major depressive disorder among a large sample of U.S. cancer survivors. Data were drawn from the 2023 and 2024 Behavioral Risk Factor Surveillance System. The analytic sample included 70,620 adult cancer survivors (56.3% female) with depression as the outcome variable. Functional limitations, loneliness, and social/emotional support were primary explanatory variables. Multivariable logistic regression models estimated adjusted odds ratios (AORs) and 95% confidence intervals (CIs), controlling for demographic, socioeconomic, and health-related covariates. Overall, 21.8% of cancer survivors reported depression. In the fully adjusted models, loneliness (AOR = 2.72, 95% CI: 2.44–3.03), functional limitations (AOR = 2.05, 95% CI: 1.81–2.31), and lack of social/emotional support (AOR = 1.43, 95% CI: 1.27–1.62) were independently associated with higher odds of depression. Female sex, low income, cost-related forgone medical care, poor self-rated health, obesity, and current/former smoking were also significantly negatively associated with depression, while physical activity was protective. We found that functional limitations, loneliness, and inadequate social support are robust and independent associated factors of depression among cancer survivors. Screening and interventions targeting these psychosocial factors may improve mental health outcomes and enhance overall survivorship care.

## Introduction

Depression is one of the most prevalent non-communicable diseases affecting an estimated 185 million adults worldwide, imposing substantial personal, social, and economic burdens [1]. According to the Diagnostic and Statistical Manual of Mental Disorders, major depressive disorder is characterized by depressed mood and/or markedly diminished interest or pleasure in activities, occurring alongside other cognitive and somatic symptoms that cause clinically significant distress or impairment [2]. Among cancer survivors, the prevalence of depression is disproportionately high, ranging from 20% to 30% depending on cancer type, treatment stage, and assessment method [3–5]. Depression in survivorship is associated with poorer health-related quality of life [6,7], reduced adherence to follow-up care [8], increased healthcare utilization [9,10], and higher mortality [11,12]. These effects underscore the need to identify modifiable psychosocial determinants that contribute to depression in this vulnerable population.

Cancer survivorship represents a unique confluence of stressors, including the physical and cognitive sequelae of treatment, functional limitations, uncertainty regarding recurrence, and social disruption [13,14]. The stress-coping framework, posits that depression arises when stressors exceed individual coping resources [15], and this is particularly relevant for cancer survivors whose illness trajectory frequently amplifies chronic stress exposure [16]. Similarly, socioecological system theory emphasizes that individual psychological outcomes are shaped by nested layers of influence ranging from intrapersonal vulnerabilities to family, community, and societal contexts [17]. In the context of survivorship, functional limitations and social disconnection represent both proximal and distal stressors interacting with individual coping capacities to influence depression risk.

Estimates of depression among cancer survivors consistently exceed those observed in the general population [3,18,19]. Available literature indicates that the onset of depression may occur pre-diagnosis, during active treatment, or in post-treatment survivorship, and is often chronic or recurrent [20–22]. Depression risk among cancer survivors reflects a complex interplay of biological, psychosocial, socioeconomic, and behavioral factors [23]. Biological mechanisms, including neuroinflammatory responses to chemotherapy, endocrine dysregulation, and pain pathways, have been implicated [24–26]. Psychosocial determinants such as loneliness [27,28], functional impairment [29], inadequate social support [30], social deprivation and financial strain [31,32], and prior psychiatric history [33] are equally critical and have all been found to significantly elevate depression risk among cancer survivors. These psychosocial factors are particularly salient given that they are modifiable, providing targets for intervention in clinical and community settings.

The extant literature indicates that functional limitations are common among cancer survivors, particularly older adults and those with treatment-related sequelae [34]. Surgical interventions, chemotherapy, radiation therapy, and hormonal treatments may result in fatigue, neuropathy, pain, reduced mobility, and cognitive impairment [35,36]. These conditions can restrict daily activities and reduce independence. Functional limitations among survivors may contribute to depression through multiple mechanisms. First, loss of autonomy and diminished participation in meaningful roles can undermine self-efficacy and identity. Second, functional limitations may increase reliance on caregivers, potentially altering social dynamics and generating feelings of burden or guilt [37]. Third, functional limitations may reduce opportunities for social interaction and physical activity, both of which are protective against depression [29,38–40]. While previous studies have documented associations between poor physical health status and depressive symptoms in cancer populations [41,42], few studies have examined functional limitations as an independent factor associated with clinically diagnosed depression in large, population-based samples. Moreover, it remains unclear whether the association between functional limitations and depression persists after adjustment for socioeconomic status and health behaviors.

Loneliness is one of the most robust psychosocial factors associated with depression across populations, including cancer survivors [27,43,44]. Loneliness reflects a subjective discrepancy between desired and actual social relationships, and differs from being alone [45]. Cancer survivors may be especially vulnerable to loneliness due to social withdrawal [46], stigma [47–49], physical limitations [50], or changes in social roles [27]. Loneliness has also been linked to dysregulation of stress-response systems, inflammatory processes, and maladaptive cognitive patterns, including negative self-appraisals and hopelessness [51,52]. These pathways may amplify vulnerability to depression.

In contrast, social and emotional support is generally conceptualized as a protective factor [53]. Supportive relationships may mitigate stress, enhance coping capacity, and provide practical assistance in coping with posttraumatic stress [54]. However, the independent effects of perceived social and emotional support relative to loneliness remain an area of ongoing investigation. Some evidence suggests that loneliness may be more strongly associated with depression than objective measures of social network size or support availability [55,56]. In cancer survivorship research, studies have often examined social support or loneliness separately [27,28]. Few have simultaneously evaluated both constructs within multivariable models that also account for functional limitations and socioeconomic constraints. Understanding their relative contributions is essential for informing targeted interventions.

Beyond psychosocial factors, depression risk is shaped by socioeconomic status (SES), healthcare access, and health behaviors. Financial strain, as reflected in low income or forgone medical care due to cost, may exacerbate psychological distress by limiting access to treatment, medications, and supportive services [57–59]. Health behaviors such as cigarette smoking, alcohol use, obesity, and physical inactivity are all established risk factors for cancer incidence [60–65]. A growing body of research also indicates that these factors may continue to play a significant role after a cancer diagnosis, shaping disease progression as well as overall health, quality of life, and depression, potentially through behavioral, inflammatory, and psychosocial pathways [19,66–69]. Studies have also found physical activity to be protective against depression among cancer survivors [70,71]. Among cancer survivors, cost-related barriers to care may carry additional emotional weight, given ongoing surveillance, treatment side effects, and fear of recurrence [72]. The intersection of financial toxicity and psychological distress is increasingly being recognized in oncology [73,74]. Therefore, disentangling the psychosocial factors associated with depression requires careful adjustment for SES and health-related factors to avoid overestimating associations attributable to confounding.

Despite robust evidence linking functional impairment [29], inadequate social support [30], and loneliness [27,28] to depression among cancer survivors, several gaps remain. Prior studies are often limited by small samples or single-site recruitment. Moreover, few studies have examined these factors together in a sample of cancer survivors, thereby limiting generalizability and the ability to assess the relative contribution of each factor. To the authors’ knowledge, this is one of the first large scale studies to examine the associations of functional limitations, loneliness, and perceived social and emotional support with self-reported lifetime diagnosis of depressive disorder among U.S. cancer survivors. Guided by stress-coping and ecological systems theory, the objectives of the present study are: 1) to estimate the prevalence of self-reported lifetime diagnosis of depressive disorder among a large sample of cancer survivors, 2) to examine bivariate associations between functional limitations, loneliness, social and emotional support, and self-reported lifetime diagnosis of depressive disorder, and 3) to assess whether these psychosocial factors remain independently associated with self-reported lifetime diagnosis of depressive disorder after sequential adjustment for sociodemographic characteristics, SES indicators, and health-related factors.

## Materials and methods

### Data source and sample

This study utilized publicly available data from the Behavioral Risk Factor Surveillance System (BRFSS), an annual cross-sectional survey administered by state health departments in collaboration with the Centers for Disease Control and Prevention (CDC). The BRFSS collects data from non-institutionalized adults aged 18 years and older residing in the U.S. and its territories through state-based telephone interviews. The survey consists of three primary components and captures data on health behaviors, chronic conditions, preventive service use, and social determinants of health (SDOH). Detailed information regarding the BRFSS sampling framework, weighting procedures, and data collection methodology is available elsewhere. The BRFSS study protocol was approved by the CDC’s Institutional Review Board (IRB), and all resulting de-identified data are publicly available. The current study was exempt from IRB review by the lead author’s institution as the data had already been de-identified. The publicly available BRFSS datasets contain no personally identifiable information and were stored electronically on password-protected, access-controlled computers accessible only to the lead author to maintain data security. The present study adhered to the Strengthening the Reporting of Observational Studies in Epidemiology (STROBE) guidelines for reporting observational research [75].

Data from the 2023 and 2024 BRFSS survey cycles were pooled to enhance statistical power for examining associations among functional limitations, social and emotional support, loneliness, and self-reported lifetime diagnosis of depressive disorder among cancer survivors. Both survey years employed comparable sampling designs and data collection procedures. Respondents were eligible for inclusion if they were aged ≥18 years, participated in a state or territory that administered the SDOH module, and reported a history of cancer as defined below. Respondents who did not meet these eligibility criteria were excluded, and observations with missing data on most analytic variables were subsequently excluded through complete-case analysis as described below. The analytic sample was restricted to respondents aged 18 years and older from states and territories (including Puerto Rico) that administered the SDOH module in 2023 and 2024 and had complete data on the variables of interest. Cancer survivorship status was defined in accordance with the National Cancer Institute’s definition, which considers an individual a cancer survivor from the time of diagnosis through the remainder of life, including those living with cancer and those free of cancer [76]. Cancer survivor status was operationalized using two items from the BRFSS: 1) “(Ever told) you had skin cancer that is not melanoma?”, and 2) “(Ever told) you had any other types of cancer?” These questions follow the stem: “Has a doctor, nurse, or other health professional ever told you that you had any of the following?” Response options include: (1) Yes, (2) No, and (7/9) Don’t know/Refused. Respondents who answered “Yes” to either item were classified as having a history of cancer. The BRFSS does not differentiate between individuals currently undergoing treatment and those in remission. Therefore, cancer survivorship status reflects a lifetime history of diagnosis rather than stage of treatment. Across both survey years, 421,585 respondents completed the SDOH module. Of these, 73,688 (17.5%) reported a history of cancer diagnosis. The overall proportion of missing data across analytic variables was 4.2%. Complete case analysis was used for observations with missing data on most analytic variables, yielding a final analytic sample of 70,620 cancer survivors. However, to minimize case loss and preserve sample size, missing responses for health insurance coverage (2.1%), body mass index (BMI; 4.5%), and household income (17.7%) were retained in analyses by coding missing responses as a separate category.

### Outcome variable

The main outcome variable examined in this study was a self-reported lifetime diagnosis of depressive disorder, and was measured as a binary variable based on response to the question, “Has a doctor, nurse, or other health professional ever told you that you have a depressive disorder (including depression, major depression, dysthymia, or minor depression)?” Respondents who answered “yes” were coded as 1, whereas respondents who answered “no” were coded as 0. Because the item assesses whether respondents had “ever” received such a diagnosis, the timing of the depressive disorder relative to the cancer diagnosis could not be determined**.**

### Explanatory variables

The main explanatory variables examined in this study were functional limitations, social and emotional support, and loneliness. Functional limitations were measured using a composite score derived from three items in the disability section of the BRFSS questionnaire. Respondents were asked whether they had difficulty: 1) walking or climbing stairs; 2) dressing or bathing; and 3) doing errands alone such as visiting a doctor’s office or shopping because of a physical, mental, or emotional condition. Respondents who indicated “yes” to any of the three items were classified as having functional limitations and were coded as 1, and respondents who indicated “no” to all three items were coded as 0. Social and emotional support was assessed using an item from the SDOH module that asked respondents, “How often do you get the social and emotional support you need?” with the following response options: “always,” “usually,” “sometimes,” “rarely,” and “never.” Respondents who indicated “always” or “usually” were considered to have adequate support and were recoded as 0, whereas respondents who indicated “sometimes,” “rarely,” or “never” were categorized as inadequate support and were recoded as 1. Loneliness was measured as a binary variable based on the question, “How often do you feel lonely?” with the following response options: “always,” “usually,” “sometimes,” “rarely,” and “never.” Respondents who indicated “rarely” or “never” were recoded as 0, whereas respondents who indicated “always,” “usually,” “sometimes” were recoded as 1. The categorization of loneliness and social/emotional support was informed by previously published studies using the same or comparable measures [77,78]. Functional limitations were categorized as present if respondents reported at least one of the three assessed limitations and absent if they reported none.

### Covariates

Covariates examined in this study were grouped under SES (education, income, health insurance coverage, and cost-related forgone medical care), poor physical health, and health risk behaviors (binge drinking, current cigarette smoking, BMI, and physical activity). These covariates were chosen based on prior research demonstrating their association with self-reported lifetime diagnosis of depressive disorder [14,18,27]. Detailed information on how each covariate was measured, and the exact questionnaire wording is provided in Supplementary S1 Table.

### Demographic variables

The following sociodemographic characteristics (survey year, age, sex, race/ethnicity, and marital status) were also included as control variables. Age was measured as an ordinal variable into the following categories “0 = 18-49 years,” “1 = 50-59 years,” “2 = 60-69 years,” “ 3 = 70-79 years,” and “4 = 80 years and above.” Adults aged 18–49 years were combined because younger cancer survivors constituted a relatively smaller proportion of the analytic sample, whereas 10-year categories were retained at older ages to capture age-related variation while maintaining adequate group sizes. Sex of the respondent was intentionally coded as a binary variable (male or female), consistent with the BRFSS sex variable used in the present analysis, with male serving as the reference category. Race/ethnicity was measured as a nominal variable into the following categories: “0 = Non-Hispanic White,” “1 = Non-Hispanic Black,” “2 = Hispanic,” and “3 = Other race/ethnicity.” Marital status was measured as a nominal variable into the following categories: “0 = Married”, “1 = Never married,” “2 = Widowed/Divorced/Separated”, and “3 = Member of an unmarried couple.”

### Data analyses

Data were analyzed using descriptive, bivariate, and multivariable analytical techniques. Descriptive statistics, including frequencies and weighted percentages, were first used to examine the distribution of all study variables. Bivariate associations between self-reported lifetime diagnosis of depressive disorder and sample characteristics were assessed using Pearson’s chi-square tests. Prior to estimating the fully adjusted model, multicollinearity among functional limitations, loneliness, and social/emotional support was assessed. Although modest correlations were observed, there was no evidence of problematic multicollinearity. The main analysis involves the use of binary logistic regression to examine associations between functional limitations, social and emotional support, loneliness, and self-reported lifetime diagnosis of depressive disorder. Four binary logistic regression models were fitted hierarchically. Model 1 adjusted for sociodemographic variables; Model 2 additionally incorporated socioeconomic status (SES) variables; Model 3 further adjusted for physical health status and health risk behaviors; and Model 4 (fully adjusted) included all previously entered variables along with functional limitations, social and emotional support, and loneliness. We also tested all pairwise two-way interactions among the three primary explanatory variables, and found that none was statistically significant (all *p* > .05). The interaction terms were therefore not retained in the final model.

The proportion of variance in self-reported lifetime diagnosis of depressive disorder explained by the set of factors was assessed using the pseudo R-square. The predictive performance of the model was estimated using the area under the receiver operating characteristic (ROC) curve for binary outcomes. Generally, the area under the ROC curve (AUC) ranges from 0.5 to 1.0, with a value of 1 indicating a perfectly fitting model, whereas values close to 0.5 indicate that the model performs no better than chance. Predicted probabilities for each model (Models 1–4) were generated from survey-weighted logistic regression using Stata’s svy: logistic command. Given the relatively large sample, ROC and AUC values were computed from unweighted predicted probabilities, with sensitivity analyses using survey-weighted ROC yielding nearly identical results, confirming robustness. Adjusted odds ratios (AORs) and 95% confidence intervals (CIs) were reported for all models. All analyses were two-tailed with statistical significance set at *p* < .05. To account for the complex sampling design and weighting of the BRFSS, Stata’s “svyset” command was applied, and all analyses were conducted using Stata MP 17.

## Results

### Sample characteristics

Table 1 presents the general distribution of the sample characteristics among cancer survivors. Of the 70,620 cancer survivors included in the analysis, 21.8% reported lifetime diagnosis of depressive disorder. A little over one in four cancer survivors had at least one functional limitation and 3.7% had all three functional limitations, 26.6% reported feeling lonely, and 18.3% reported lacking social and emotional support. About half of the sample were aged 70 years and above (47.2%), and the majority (56.3%) were female. The majority of the respondents self-identified as non-Hispanic White (80.9%), followed by Hispanic (7.6%), non-Hispanic Black (5.2%), and other race/ethnicity (6.3%). Slightly over 60% of respondents were married, 29.1% were Widowed/Divorced/Separated, and 7.4% had never been married. A little over one-third of respondents (37.1%) had graduated from college or technical school, 33.8% had attended some college or technical school, and 29.1% had a high school education or less. More than one in three respondents (38.1%) reported an annual household income of $75,000 or higher, whereas 10.6% reported income below $25,000. About 6% of cancer survivors had cost-related forgone medical care in the past 12 months. More than one in four respondents (27.7%) rated their physical health as poor, and 8.5% engaged in binge drinking. Regarding smoking status, 55% had never smoked cigarettes, 35.4% were former smokers, and 9.6% currently smoke cigarettes. With respect to BMI, 35% were classified as overweight, 30.3% as obese, and 29.8% as having normal weight. About three in four respondents (73.4%) reported being physically active.

**Table 1:** Respondents’ characteristics stratified by depression among cancer survivors (n = 70,620).

Variables	Unweighted Frequency (Weighted %)	Major Depressive Disorder		Chi-square (sig)
		% No	% Yes	
**Outcome variable**				
Diagnosis of Depression				
No	56,184 (78.2)			
Yes	14,436 (21.8)			
**Explanatory variables**				
**Sociodemographic factors**				
Survey Year				0.10 (*p* = .750)
2023	36,704 (53.4)	79.5	20.5	
2024	33,916 (46.6)	79.6	20.4	
Age				2,318.45 (*p* < .001)
18-49 years	4,913 (11.7)	62.3	37.7	
50-59 years	7,554 (13.9)	70.1	29.9	
60-69 years	18,039 (27.2)	76.9	23.1	
70-79 years	24,709 (30.2)	82.0	18.0	
80 years and above	15,405 (17.0)	88.9	11.1	
Sex				1,267.81 (*p* < .001)
Male	31,241 (43.7)	85.6	14.4	
Female	39,379 (56.3)	74.7	25.3	
Race/ethnicity				107.34 (*p* < .001)
Non-Hispanic Whites	63,251 (80.9)	80.0	20.0	
Non-Hispanic Blacks	2,297 (5.2)	80.5	19.5	
Hispanics	2,330 (7.6)	73.4	26.6	
Other	2,742 (6.3)	734.4	25.6	
Marital status				843.91 (*p* < .001)
Married	40,818 (61.4)	83.2	16.8	
Never married	4,759 (7.4)	71.2	28.8	
Widowed/Divorced/Separated	23,620 (29.1)	75.6	24.4	
Member of unmarried couple	1,423 (2.2)	69.7	30.3	
**SES Factors**				
Highest level of education				120.60 (*p* < .001)
High school or less	17,069 (29.1)	78.2	21.8	
Attended some college or technical school	19,336 (33.8)	77.7	22.3	
Graduated from college or technical school	34,215 (37.1)	81.3	18.7	
Income				1,543.08 (*p* < .001)
Less than $25,000	7,176 (10.6)	64.2	35.8	
$25,000-$34,999	6,377 (8.7)	73.7	26.3	
$35,000-$49,999	8,495 (10.9)	77.9	22.1	
$50,000-$74,999	10,740 (14.0)	81.0	19.0	
$75,000 and above	25,574 (38.1)	83.4	16.6	
Missing	12,258 (17.7)	83.3	16.7	
Has health insurance				73.98 (*p* < .001)
Yes	68,485 (96.4)	79.6	20.4	
No	706 (1.5)	67.3	32.7	
Missing	1,429 (2.1)	82.6	17.4	
Cost-related forgone medical care				1424.00 (*p* < .001)
No	67,439 (94.2)	80.8	19.2	
Yes	3,181 (5.8)	53.2	46.8	
Self-rated health				2,314.62 (*p* < .001)
Good	52,422 (72.3)	83.9	16.1	
Poor	18,198 (27.7)	67.2	32.8	
**Health risk behaviors**				
Binge drinking				34.34 (*p* < .001)
No	65,598 (91.5)	79.8	20.2	
Yes	5,022 (8.5)	76.3	23.7	
Current smoking status				1046.72 (*p* < .001)
Never smoke	39,042 (55.0)	82.4	17.6	
Former smoker	25,698 (35.4)	78.7	21.3	
Currently smoke	5,880 (9.6)	64.3	35.7	
BMI				639.99 (*p* < .001)
Normal	21,732 (29.8)	81.9	18.1	
Overweight	25,373 (35.5)	82.3	17.7	
Obese	20,505 (30.3)	73.6	26.4	
Missing	3,010 (4.5)	79.9	20.1	
Physical activity				762.15 (*p* < .001)
Inactive	18,141 (26.6)	72.4	27.6	
Active	52,479 (73.4)	82.0	18.0	
**Functional limitations**				
Functional limitations				2818.49 (*p* < .001)
None	51,886 (72.7)	83.9	16.1	
One limitation	12,291 (17.3)	72.1	27.9	
Two limitations	4,201 (6.3)	62.0	38.0	
Three limitations	2,242 (3.7)	52.6	47.4	
**Social and Emotional Support and Loneliness**				
Has social and emotional support				2,602.92 (*p* < .001)
Yes	58,867 (81.7)	83.0	17.0	
No	11,753 (18.3)	62.2	37.8	
Loneliness				5330.96 (*p* < .001)
No	52,468 (73.4)	86.1	13.9	
Yes	18,152 (26.6)	60.7	39.3	

SES = Socioeconomic Status; BMI = Body Mass Index.

Except for survey year, all variables examined were significantly associated with depression at the bivariate level. For instance, the prevalence of depression among respondents who reported feeling lonely (39.3%) was significantly greater than among those who did not report loneliness (13.9%; χ^2^(1) = 79.36, *p* < .001). Similarly, 37.8% of respondents who lacked social and emotional support had depression, compared to 17.0% of those reporting adequate social and emotional support (χ^2^(1) = 2602.92, *p* < .001). About one in three respondents with functional limitations (32.5%) had depression compared to 16.1% of respondents without functional limitations who had depression (χ^2^(1) = 2270.48, *p* < .001).

### Multivariable logistic regression results

Table 2 presents results from the multivariable binary logistic regression analysis examining the association between functional limitations, social and emotional support, and loneliness and self-reported lifetime diagnosis of depressive disorder among cancer survivors. We found a significant association between functional limitations, social and emotional support, and loneliness and self-reported lifetime diagnosis of depressive disorder, independent of sociodemographic, socioeconomic, physical health, and behavioral factors. In Model 4, respondents who reported feeling lonely had 2.72 times higher odds of having lifetime diagnosis of depressive disorder when compared to their counterparts who did not report feeling lonely (*AOR* = 2.72; 95% CI = 2.44–3.03; *p* < .001). Compared to respondents with no functional limitations, those with at least one functional limitation had double the odds of having lifetime diagnosis of depressive disorder (*AOR* = 2.05; 95% CI = 1.81–2.31; *p* < .001). Additionally, respondents who lacked social and emotional support had 1.43 times higher odds of having lifetime diagnosis of depressive disorder when compared to respondents who had adequate social and emotional support (*AOR* = 1.43; 95% CI = 1.27–1.62; *p* < .001).

**Table 2:** Multivariable binary logistic regression examining emotional support and loneliness as factors associated with self-reported lifetime diagnosis of depressive disorder among cancer survivors (n = 70,620).

Variables	Model 1: sociodemographic factors		Model 2: sociodemographic factors + SES		Model 3: sociodemographic factors + SES + HB		Model 4 (Fully Adjusted Model)	
	AOR (95% C.I.)	*p*-value	AOR (95% C.I.)	*p*-value	AOR (95% C.I.)	*p*-value	AOR (95% C.I.)	*p*-value
**Sociodemographic factors**								
Survey year (2023)								
2024	1.05 (0.96-1.14)	.301	1.06 (0.97-1.16)	.225	1.05 (0.96-1.15)	.255	1.05 (0.96-1.16)	.298
Age (18–49 years)								
50-59 years	0.78 (0.68-0.90)	.001	0.81 (0.69-0.94)	.005	0.74 (0.63-0.86)	<.001	0.77 (0.65-0.91)	.002
60-69 years	0.61 (0.53-0.70)	< .001	0.61 (0.52-0.71)	< .001	0.56 (0.48-0.65)	< .001	0.56 (0.48-0.66)	< .001
70-79 years	0.48 (0.42-0.55)	< .001	0.49 (0.42-0.57)	< .001	0.46 (0.39-0.53)	< .001	0.47 (0.40-0.55)	< .001
80 years and above	0.21 (0.18-0.25)	< .001	0.22 (0.19-0.26)	< .001	0.21 (0.18-0.25)	< .001	0.20 (0.16-0.24)	< .001
Sex (Male)								
Female	1.97 (1.81-2.15)	< .001	1.94 (1.78-2.12)	< .001	2.15 (1.96-2.36)	< .001	2.15 (1.95-2.37)	< .001
Race/ethnicity (Non-Hispanic Whites)								
Non-Hispanic Blacks	0.82 (0.59-0.87)	.001	0.65 (0.54-0.79)	<.001	0.58 (0.47-0.70)	<.001	0.54 (0.44-0.65)	<.001
Hispanics	1.06 (0.83-1.36)	.654	0.90 (0.68-1.18)	.435	0.84 (0.64-1.12)	.231	0.84 (0.63-1.14)	.263
Other	1.20 (0.92-1.56)	.189	1.10 (0.84-1.43)	.495	1.02 (0.78-1.34)	.882	0.85 (0.64-1.13)	.265
Marital status (Married)								
Never married	1.49 (1.28-1.74)	<.001	1.22 (1.04-1.43)	.016	1.22 (1.03-1.45)	.018	1.06 (0.89-1.27)	.502
Widowed/Divorced/Separated	1.72 (1.56-1.90)	< .001	1.36 (1.22-1.52)	< .001	1.30 (1.16-1.45)	< .001	1.03 (0.92-1.17)	.593
Member of unmarried couple	1.65 (1.30-2.09)	<.001	1.42 (1.11-1.80)	.005	1.42 (1.10-1.83)	.007	1.30 (0.99-1.71)	.060
**SES factors**								
Highest level of education (High school or less)								
Attended college or technical school			1.04 (0.92-1.17)	.547	1.18 (1.04-1.33)	.009	1.20 (1.05-1.36)	.006
Graduated from college or technical school			0.97 (0.87-1.09)	.661	1.31 (1.17-1.48)	< .001	1.36 (1.20-1.54)	< .001
Income (Less than $25,000)								
$25,000-$34,999			0.76 (0.63-0.91)	.003	0.87 (0.71-1.05)	.141	0.92 (0.76-1.13)	.435
$35,000-$49,999			0.67 (0.56-0.80)	< .001	0.79 (0.65-0.95)	.011	0.92 (0.76-1.10)	.363
$50,000-$74,999			0.62 (0.51-0.76)	< .001	0.80 (0.66-0.99)	.037	0.93 (0.75-1.15)	.499
$75,000 and above			0.45 (0.38-0.54)	< .001	0.65 (0.54-0.78)	< .001	0.80 (0.66-0.97)	.024
Missing			0.53 (0.44-0.64)	< .001	0.68 (0.55-0.83)	< .001	0.77 (0.62-0.94)	.011
Health insurance (Yes)								
No			0.67 (0.47-0.95)	.023	0.74 (0.52-1.05)	.094	0.73 (0.51-1.05)	.089
Missing			0..90 (0.66-1.23)	.512	0.92 (0.68-1.24)	.581	0.93 (0.70-1.25)	.641
Cost-related forgone medical care (No)								
Yes			2.11 (1.77-2.50)	< .001	1.74 (1.46-2.08)	< .001	1.33 (1.11-1.60)	.002
**Health and health behaviors**								
Self-rated health (Good)								
Poor					2.09 (1.87-2.34)	< .001	1.48 (1.31-1.67)	< .001
Binge drinking (No)								
Yes					0.94 (0.82-1.07)	.341	0.95 (0.82-1.09)	.451
Cigarette smoking (Never smoked)								
Former smoker					1.47 (1.32-1.63)	< .001	1.45 (1.30-1.62)	< .001
Current smoker					1.69 (1.44-1.98)	< .001	1.58 (1.35-1.85)	< .001
BMI (Normal)								
Overweight					1.06 (0.95-1.19)	.316	1.08 (0.95-1.22)	.232
Obese					1.36 (1.21-1.52)	< .001	1.30 (1.15-1.46)	< .001
Missing					0.94 (0.73-1.20)	.602	0.92 (0.71-1.19)	.524
Physical activity (Inactive)								
Active					0.69 (0.62-0.77)	< .001	0.84 (0.74-0.94)	.003
**Functional limitations**								
Functional limitations (None)								
At least one							2.05 (1.81-2.31)	< .001
Emotional Support and Loneliness								
Has social and emotional support (Yes)								
No social and emotional support							1.43 (1.27-1.62)	< .001
Loneliness (No)								
Yes							2.72 (2.44-3.03)	< .001
**Model fitness indices**								
AUC	0.6683		0.6859		0.7181		0.7621	
Pseudo R-square	0.0596		0.0771		0.1059		0.1558	

Abbreviations: AOR = Adjusted Odds Ratio; CI = Confidence Interval; SES = Socioeconomic Status; BMI = Body Mass Index.

Apart from survey year, all sociodemographic variables included in Model 1 were significantly associated with self-reported lifetime diagnosis of depressive disorder. These associations largely remained in Models 2, 3, and 4. However, marital status was no longer significant in the fully adjusted model (Model 4) after accounting for functional limitations, social and emotional support, and loneliness. In Model 4, we found that age was inversely associated with self-reported lifetime diagnosis of depressive disorder, with older respondents less likely to report lifetime diagnosis of depressive disorder. Females had more than twice the odds of self-reported lifetime diagnosis of depressive disorder compared to males (*AOR* = 2.15; 95% CI = 1.98–2.37; *p* < .001). Compared to non-Hispanic White respondents, non-Hispanic Black respondents had 46% lower odds of self-reported lifetime diagnosis of depressive disorder (*AOR* = 0.54; 95% CI = 0.44–0.65; *p* < .001).

Education was not significantly associated with self-reported lifetime diagnosis of depressive disorder in Model 2, but became significant in Models 3 and 4. In the fully adjusted Model, respondents who graduated from college or technical school (*AOR* = 1.36; 95% CI = 1.20–1.54; *p* < .001) or attended college or technical school (*AOR* = 1.20; 95% CI = 1.05–1.36; *p* = .006) were more likely to report lifetime diagnosis of depressive disorder when compared to respondents with high school or less education. Household income and cost-related forgone medical care were significantly associated with self-reported lifetime diagnosis of depressive disorder in Models 2–4. After full adjustment in Model 4, respondents with income of $75,000 or more had 20% lower odds of reporting lifetime diagnosis of depressive disorder (*AOR* = 0.80; 95% CI = 0.66–0.97; *p* = .024). Respondents who reported cost-related forgone medical care had 1.33 times higher odds of having lifetime diagnosis of depressive disorder (*AOR* = 1.33; 95% CI = 1.11–1.60; *p* = .002).

Among health status and behavioral factors, poor self-rated health, cigarette smoking, BMI, and physical activity were significantly associated with self-reported lifetime diagnosis of depressive disorder in Models 3 and 4. In the fully adjusted model, respondents with poor self-rated health had higher odds of self-reported lifetime diagnosis of depressive disorder (AOR = 1.48; 95% CI = 1.31–1.67; *p* < .001). Former smokers (AOR = 1.45; 95% CI = 1.30–1.62; *p* < .001) and current smokers (AOR = 1.58; 95% CI = 1.35–1.85; *p* < .001) had greater odds of self-reported lifetime diagnosis of depressive disorder compared to never smokers. Obesity was also associated with increased odds of self-reported lifetime diagnosis of depressive disorder (AOR = 1.30; 95% CI = 1.15–1.46; *p* < .001). In contrast, physical activity was protective; physically active respondents had 16% lower odds of self-reported lifetime diagnosis of depressive disorder compared to inactive respondents (AOR = 0.84; 95% CI = 0.74–0.94; *p* = .003).

### Model fit and predictive performance

Model fit statistics indicated progressive improvement across the hierarchical models. Based on the pseudo r-square statistic, sociodemographic variables in Model 1 explained 5.96% of the variance in self-reported lifetime diagnosis of depressive disorder. The addition of SES variables in Model 2 increased the explained variance to 7.71%. Incorporating physical health status and health behaviors in Model 3 further increased the explained variance to 10.59%. The fully adjusted Model 4 explained 15.58% of the variance in self-reported lifetime diagnosis of depressive disorder. ROC curve analyses demonstrated improved classification accuracy across models. Figure 1A shows that Model 1 yielded an area under the curve (AUC) of 0.6683 and correctly classified 66.83% of respondents. Model 2 improved classification to 68.59% (AUC = 0.6859; Fig 1B). Model 3 achieved 71.81% correct classification (AUC = 0.7181; Fig 1C). The fully adjusted Model 4 demonstrated the strongest predictive performance, correctly classifying 76.21% of respondents (AUC = 0.7621; Fig 1D).

**Fig 1:**
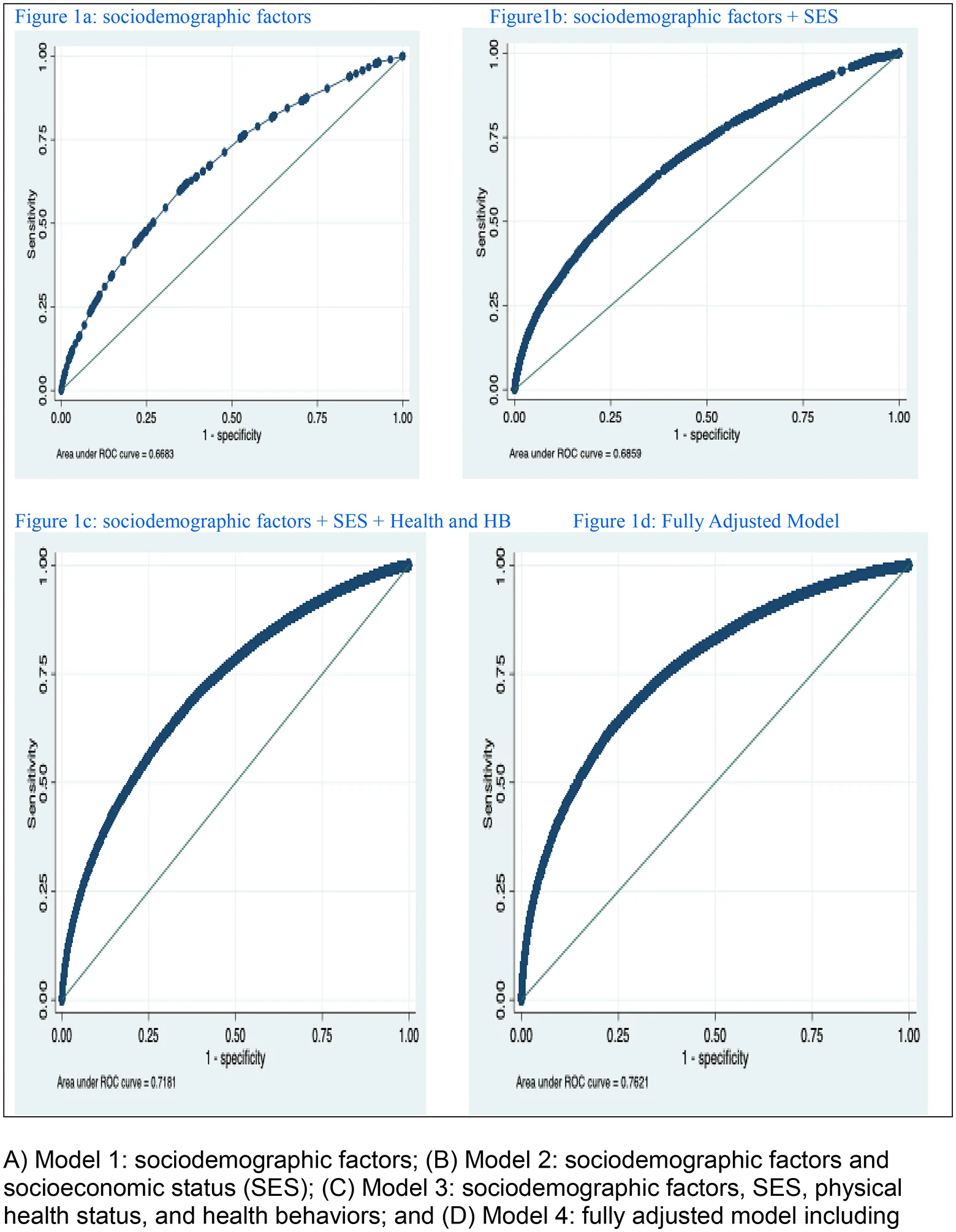
ROC curves for the hierarchical logistic regression models predicting self-reported lifetime diagnosis of depressive disorder among cancer survivors. (A) Model 1: sociodemographic factors; (B) Model 2: sociodemographic factors and socioeconomic status (SES); (C) Model 3: sociodemographic factors, SES, physical health status, and health behaviors; and (D) Model 4: fully adjusted model including sociodemographic factors, SES, physical health status, health behaviors, functional limitations, social and emotional support, and loneliness.

## Discussion

In this large sample of cancer survivors, loneliness, functional limitations, and inadequate social and emotional support were independently associated with self-reported lifetime diagnosis of depressive disorder. We found that 21.8% of cancer survivors self-reported lifetime diagnosis of depressive disorder, confirming that depression remains a substantial public health burden in survivorship. After sequential adjustment for sociodemographic, socioeconomic, and health-related characteristics, loneliness, functional limitations, and inadequate social and emotional support remained independently associated with self-reported lifetime diagnosis of depressive disorder. Loneliness was the strongest correlate (AOR = 2.72), followed by functional limitations (AOR = 2.05) and inadequate social and emotional support (AOR = 1.43). The incremental improvement in model fit and discrimination (AUC increasing to 0.7621) underscores the explanatory contribution of psychosocial variables beyond traditional demographic and clinical factors. Taken together, these findings support the conceptualization of depression in cancer survivorship as a stress-mediated outcome shaped by functional impairment and social disconnection. Psychosocial vulnerabilities were not secondary correlates but strong determinants, persisting independent of socioeconomic position, health behaviors, and self-rated health.

Loneliness emerged as the strongest factor associated with self-reported lifetime diagnosis of depressive disorder, suggesting it functions as a primary psychosocial pathway linking cancer-related stress to depressive outcomes. Survivors may experience loneliness despite the presence of social networks, particularly if illness-related changes disrupt role fulfillment or relational reciprocity [27,28]. Mechanistically, loneliness may amplify cognitive vulnerabilities such as rumination [79] and perceived burdensomeness [80], while also activating biological stress pathways implicated in depression, including dysregulation of the hypothalamic-pituitary-adrenal axis and inflammatory processes [25,81]. Other scholars have also found that social connectedness may mediate the relationship between loneliness and depression, highlighting connectedness as a potential intervention target for reducing depressive symptoms among cancer survivors [82]. Beyond depressive symptoms, broader research suggests that loneliness among cancer survivors is associated with poorer overall well-being and may contribute to worse survival outcomes [83]. Furthermore, structured social and behavioral interventions have been found to reduce loneliness and improve psychological outcomes among survivors [84,85], making it a compelling target for clinical and community-based strategies aimed at mitigating depression risk.

Functional limitations independently increased self-reported lifetime diagnosis of depressive disorder, consistent with the stress-process framework [86]. Beyond restricting physical activity, functional limitations may undermine role engagement and identity coherence, which are central to psychosocial resilience [87]. Cancer survivorship guidelines increasingly emphasize functional assessment and rehabilitation as essential components of comprehensive care [88]. Integrating rehabilitation and adaptive interventions into survivorship care may therefore improve both physical and psychological outcomes.

Adequate social and emotional support reduced the odds of self-reported lifetime diagnosis of depressive disorder, highlighting the protective role of relational resources in survivorship [30,89,90]. Although survivorship marks the completion of active treatment for many individuals, it often introduces ongoing psychosocial challenges, including persistent physical symptoms, uncertainty about recurrence, and disruptions in social roles. Inadequate social and emotional support has been linked not only to depression and anxiety in early survivorship but also to stronger associations between physical symptoms such as fatigue, suggesting that inadequate social and emotional support may exacerbate psychological vulnerabilities even after treatment ends [29]. Notably, structural indicators of social connection, such as marital status, were attenuated once subjective experiences of loneliness and social and emotional support were taken into account. This distinction is consistent with research demonstrating that perceived support is more predictive of mental health outcomes than structural relationship status alone [91,92]. Given the established links between depression and diminished quality of life in survivorship, interventions designed to enhance social support and connection may represent a modifiable pathway to improving long-term psychosocial outcomes in this growing population. Specifically, depression may contribute to social withdrawal and greater feelings of loneliness, perceptions of inadequate social and emotional support, and functional impairment. Thus, the observed associations should not be interpreted as indicating that functional limitations, loneliness, or inadequate social/emotional support necessarily preceded depression.

Female sex was significantly associated with higher odds of self-reported lifetime diagnosis of depressive disorder, consistent with longstanding epidemiologic evidence demonstrating greater depression prevalence among women [62]. Older age appeared protective, aligning with some survivorship studies indicating lower depressive symptom burden among older adults compared with younger survivors [3]. Higher household income attenuated self-reported lifetime diagnosis of depressive disorder, whereas cost-related forgone care increased it, supporting evidence that financial toxicity functions as a chronic psychosocial stressor influencing mental health outcomes in oncology populations [73,74]. The association between higher educational attainment and self-reported lifetime diagnosis of depressive disorder risk may reflect greater health literacy, improved access to diagnostic services, or reporting patterns rather than a direct causal association, consistent with broader mental health detection literature [62]. Smoking and obesity were independently associated with self-reported lifetime diagnosis of depressive disorder, aligning with established behavioral health literature [61,62,64,65]. Physical activity was protective, consistent with meta-analytic evidence demonstrating the antidepressant effects of exercise, including among cancer survivors [70,71].

The findings of this study support integrating routine loneliness screening into cancer survivorship clinics, establishing standardized rehabilitation referral pathways for survivors with functional limitations, and embedding financial toxicity screening models to identify and address economic distress early. All of which are consistent with National Comprehensive Cancer Network Distress Management Guidelines [93]. Embedding these psychosocial, functional, and financial assessments within multidisciplinary survivorship care can strengthen prevention efforts for major depressive disorder and promote more equitable, whole-person oncology care, which aligns with recommendations from national survivorship frameworks [88]. Such strategies are particularly critical for socioeconomically vulnerable survivors, who face compounded psychosocial risks [73,74]. Intervention trials evaluating scalable psychosocial and functional interventions should prioritize depression as a primary endpoint.

## Limitations

The findings of this study should be interpreted in light of some limitations. First, the use of cross-sectional data precludes any causal inference and limits the ability to determine temporal ordering between psychosocial vulnerabilities and self-reported lifetime diagnosis of depressive disorder. These associations may be bidirectional. Although these psychosocial factors may be associated with self-reported lifetime diagnosis of depressive disorder, depression itself may contribute to functional impairment, greater feelings of loneliness, and reduced or perceived inadequate social and emotional support. Future longitudinal and prospective cohort studies are needed to clarify the temporal ordering and direction of these associations. Second, all measures including lifetime diagnosis of depressive disorder, functional limitations, loneliness, and social and emotional support were self-reported, raising the possibility of recall bias and misclassification. Moreover, the depressive disorder measure does not establish a current clinical diagnosis or the timing of the diagnosis relative to cancer. Thus, some respondents may have received a depressive disorder diagnosis before their cancer diagnosis. Future research should incorporate clinical diagnostic interviews and validated multi-item scales to enhance measurement precision. Third, the BRFSS lacked detailed cancer-specific information (e.g., cancer type, stage, treatment modality, and time since diagnosis), which may confound associations. Future studies should integrate oncology registry or clinical data to enable more nuanced, cancer-specific analyses. Fourth, unmeasured confounders such as prior psychiatric history, cumulative life stress exposure, and access to mental health services may have influenced the observed associations. Future research should include comprehensive psychosocial and clinical covariates to reduce residual confounding. Finally, although the sample was large, institutionalized populations and individuals without telephone access were not included, potentially limiting generalizability. Future studies that employ mixed-mode or population-based registry approaches are needed to ensure broader inclusion of medically and socially vulnerable survivors.

## Conclusion

In conclusion, loneliness, functional limitations, and inadequate social and emotional support were independently associated with self-reported lifetime diagnosis of depressive disorder among U.S. cancer survivors, underscoring the central role of psychosocial and functional vulnerabilities in survivorship mental health. Clinically, these findings highlight the need for systematic screening and integrated care models that address social disconnection and physical impairment alongside traditional oncology follow-up. At the policy level, embedding loneliness, rehabilitation, and financial distress assessments into survivorship care pathways may strengthen early identification, prevention, and equitable mental health support for cancer survivors.

## Supporting information

S1 Table List of Covariates. (DOCX)
