# Supplementary material for: Modifiable psychosocial risk factors for depression among cancer survivors in the United States
Source: PLoS One. 2026 Oct 21;21(10):e0359599. doi: 10.1371/journal.pone.0359599 (PMC13686275; doi:10.1371/journal.pone.0359599)
Supplement: Supplementary file 1 [file pone.0359599.s001.docx]

Supplementary Table 1

List of covariates

| Variable name | | Survey question(s) | Analytic coding |
| --- | --- | --- | --- |
| **Demographic characteristics** | |  |  |
|  | Survey year |  | 0 = 2023  1 = 2024 |
|  | Age |  | 0 = 18-49 years  1 = 50-59 years  2 = 60-69 years  3 = 70-79 years  4 = 80 years and above |
|  | Sex | Are you male or female? | 0 = Male  1 = Female |
|  | Race/ethnicity |  | 0 = Non-Hispanic White  1 = Non-Hispanic Blacks  2 = Hispanics  3 = Other |
|  | Marital status | Are you (marital status)? | 0 = Married  1 = Never married  2 = Widowed or Divorced or Separated  3 = Member of unmarried couple |
| **Socioeconomic status** | |  |  |
|  | Highest level of education | What is the highest grade or year of school you completed? | 0 = High school or less  1 = Attended some college or technical school  2 = Graduated from college or technical school |
|  | Income | Is your annual household income from all sources? | 0 = Less than $25,000  1 = $25,000-$34,999  2 = $35,000-$49,999  3 = $50,000-$74,999  4 = $75,000 and above  5 = Missing |
|  | Has health insurance coverage | Adults who had some form of health insurance | 0 = No  1 = Yes  2 = Missing |
|  | Cost-related forgone medical care | Was there a time in the past 12 months when you needed to see a doctor but could not because you could not afford it? | 0 = No  1 = Yes |
| **Health and Health risk behaviors** | |  |  |
|  | Self-rated health | Would you say that in general your health is? | 0 = Good  1 = Poor |
|  | Binge drinking | Considering all types of alcoholic beverages, how many times during the past 30 days did you have 5 or more drinks for men or 4 or more drinks for women on an occasion? | 0 = No  1 = Yes |
|  | Current smoking status | Have you smoked at least 100 cigarettes in your entire life?  Do you now smoke cigarettes every day, some days, or not at all? | 0 = Never  1 = Former  2 = Current |
|  | BMI classification | Classification Body Mass Index (BMI) | 0 = Normal  1 = Overweight  2 = Obese  3 = Missing |
|  | Physical activity | During the past month, other than your regular job, did you participate in any physical activities or exercises such as running, calisthenics, golf, gardening, or walking for exercise? | 0 = Inactive  1 = Active |
